# Design of New Competitive Dengue Ns2b/Ns3 Protease Inhibitors—A Computational Approach

**DOI:** 10.3390/ijms12021089

**Published:** 2011-02-09

**Authors:** Neni Frimayanti, Chin Fei Chee, Sharifuddin M. Zain, Noorsaadah Abd. Rahman

**Affiliations:** Department of Chemistry, Faculty of Science, University of Malaya, 50603 Lembah Pantai, Kuala Lumpur, Malaysia; E-Mails: nenifrimayanti@yahoo.com (N.F.); cfchee@yahoo.com (C.F.C.); smzain@um.edu.my (S.M.Z.)

**Keywords:** dengue NS2B/NS3 protease, molecular docking, interaction energy, binding energy, complexation energy

## Abstract

Dengue is a serious disease which has become a global health burden in the last decade. Currently, there are no approved vaccines or antiviral therapies to combat the disease. The increasing spread and severity of the dengue virus infection emphasizes the importance of drug discovery strategies that could efficiently and cost-effectively identify antiviral drug leads for development into potent drugs. To this effect, several computational approaches were applied in this work. Initially molecular docking studies of reference ligands to the DEN2 NS2B/NS3 serine protease were carried out. These reference ligands consist of reported competitive inhibitors extracted from *Boesenbergia rotunda* (*i.e.*, 4-hydroxypanduratin A and panduratin A) and three other synthesized panduratin A derivative compounds (*i.e.*, 246DA, 2446DA and 20H46DA). The design of new lead inhibitors was carried out in two stages. In the first stage, the enzyme complexed to the reference ligands was minimized and their complexation energies (*i.e.*, sum of interaction energy and binding energy) were computed. New compounds as potential dengue inhibitors were then designed by putting various substituents successively on the benzyl ring A of the reference molecule. These substituted benzyl compounds were then computed for their enzyme-ligand complexation energies. New enzyme-ligand complexes, exhibiting the lowest complexation energies and closest to the computed energy for the reference compounds, were then chosen for the next stage manipulation and design, which involved substituting positions 4 and 5 of the benzyl ring A (positions 3 and 4 for 2446DA) with various substituents.

## 1. Introduction

Dengue is a serious re-emerging infectious disease that is endemic in over 100 countries. The U.S. Center for Disease Control and Prevention (CDC) estimated about 2.5 billion people worldwide to be at risk for dengue infections. About 50–100 million dengue infections occur annually with approximately 500,000 cases being the dengue hemorrhagic fever (DHF) and dengue shock syndrome (DSS) causes approximately 25,000 deaths [1] yearly. Currently, there is no approved vaccine or antiviral therapy to combat this disease.

There are four serotypes of dengue virus (DEN1, DEN2, DEN3 and DEN4), with dengue virus type 2 (DEN2) being the most prevalent. This virus is a single stranded RNA of positive polarity with a type I cap structure at the 5′-end and codes for single polyprotein precursor (3391 amino acid residues for DEN2) arranged in order NH_2_-C-prM-E-NS1-NS2A-NS2B-NS3-NS4A-NS4B-NS5-COOH [2]. The *N*-terminal region of the non-structural 3 protein (NS3) is a serine protease [3,4] that binds to an NS2B cofactor which is required to cleave the polyprotein. This NS2B-NS3 protease complex is required for viral replication [5]. Thus, it serves as a promising target for dengue virus antiviral drug development [6,7].

Several compounds extracted from *Boesenbergia rotunda* (L.) Mansf. Kulturpfl (BRI), a common spice of the ginger family (Zingiberaceae), has shown inhibitory activity against the ability of DEN2 serine protease to cleave fluorogenic peptide substrates [8].

In this study, computations on the interactions at the active site of DEN2 NS2B/NS3 protease were carried out for five ligands, 4-hydroxypanduratin A, panduratin A, 246DA, 2446DA and 20H46DA. All these ligands have shown to be competitive inhibitors for the DEN2 NS2B/NS3 protease activity. 4-hydroxypanduratin A and panduratin A are natural product extracts [8] while 246DA, 2446DA and 20H46DA are compounds which were synthesized in our research group. These ligands were docked to the receptor active site and these complexes were further minimized. From the spatial arrangement, contributions from each structure of the ligand with the residues of the active site were calculated. New competitive derivatives were then designed based on the results and the structural information obtained.

## 2. Results and Discussion

### 2.1. Spatial Arrangement at the Active Site of the DEN2 NS2B/NS3 Protease

**Figure 1.**
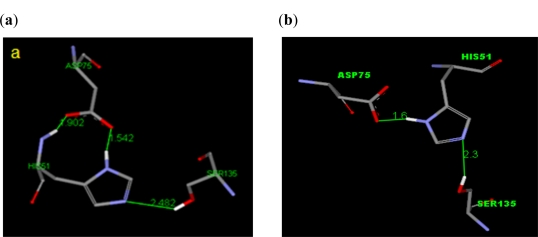
The spatial arrangement of the catalytic triad of (**a**) DEN2 NS2B/NS3 (2FOM. Pdb); (**b**) previous homology modeling [10].

Lee and his co-workers proposed a homology model to clarify the orientation of the catalytic triad in order to improve the proteolytic activity [9]. In this study, the distances between the three residues in the catalytic triad (His51, Asp75 and Ser135) of the minimized three dimensional structure of NS2B/NS3 serine protease of the DEN2 virus, were found to be close to the homology model reported by Lee and his co-workers [9]. The catalytic triad residues for DEN2 NS2B/NS3 serine protease were found to be conserved and that the 2FOM structure was suitable to be used in the next step (docking). The spatial arrangement of that catalytic triad is presented in Figure 1.

### 2.2. Competitive Inhibitors Extracted from Boesenbergia Rotunda

#### 2.2.1. Docking of 4-Hydroxypanduratin A and Panduratin A to DEN2 NS2B/NS3

**Figure 2.**
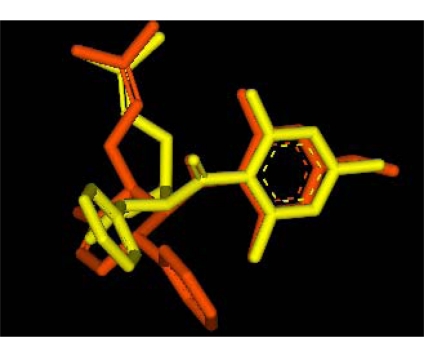
Superimposition of the competitive inhibitor structures, 4-hydroxypanduratin A (yellow) and panduratin A (orange).

Two competitive dengue inhibitor compounds were docked onto the serine protease enzyme. Figure 2 shows the superimposition of these two compounds (*i.e.*, 4-hydroxypanduratin A and panduratin A). Both 4-hydroxypanduratin A and panduratin A were observed to take up similar poses with similar binding orientation around the active sites of the serine protease NS2B/NS3 (Figure 2) and these ligands were observed to interact with the residues in the catalytic triad (*i.e.*, His51, Asp75 and Ser135) of the protease. Lee and co-workers reported these ligands formed a hydrogen bond with the carbonyl group of Gly151and the hydroxyl of Ser135 [10] but no interaction with the His51 of the catalytic triad.

**Figure 3.**
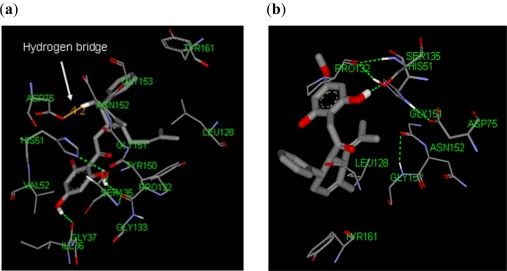
The binding site of the competitive inhibitors: (**a**) 4-hydroxypanduratin A; (**b**) panduratin A.

Our results showed 4-hydroxypanduratin A and panduratin A exhibited Van der Waals interactions with Pro132 and His51. In addition, hydrogen bonding interaction was also observed between these two ligands and the residues His51 and Gly153, indicating another possible mode of interaction between these ligands and the DEN2 NS2B/NS3 protease. These binding interactions are shown in Figure 3.

Interaction with the Gly153 was also observed from homology modeling reported by Brinkworth and co-workers [11] in which they observed the positioning of Gly153 and other residues, such as Tyr150 surrounding the binding site of DEN2 NS2B/NS3 to form a small hydrophobic specificity pocket for P1 (P1 is the substrate residue at the amino terminal). However, interactions with Tyr150 were not observed in our study.

4-hydroxypanduratin A was also found to interact with the residue Ile36 via hydrogen bonding between the carboxyl of Ile36 and hydroxyl hydrogen of the ligand. In addition, a hydrogen bridge (*i.e.*, an interaction with the distances between a hydrogen atom of a ligand and the catalytic triad residues in the binding site being less than 10 Å) with a distance of 4.2 Å between 4-hydroxypanduratin A and the carboxyl group of Asp75 was observed. A hydrogen bridge was also observed between Asp75 and panduratin A, but at a much larger distance of 6.6 Å. This may be the reason for 4-hydroxypanduratin A being more active than panduratin A.

The complexation energy for both competitive inhibitors were calculated and summarized in Table 1. 4-hydroxypanduratin A has a lower complexation energy value compared to panduratin A. This result is consistent with the inhibitory constant (K_i_) value observed experimentally for both these compounds.

#### 2.2.2. Design of New Dengue Virus Inhibitors Based on 4-Hydroxypanduratin A and Panduratin A

**Figure 4.**
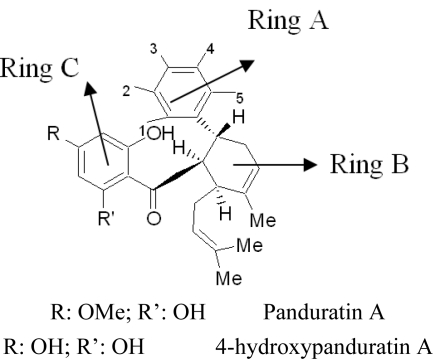
Molecular structures of competitive inhibitors extracted from *Boesenbergia rotunda.*

New compounds with potential inhibitory activities against dengue virus were then designed by adding substituents on the various positions (*i.e*., positions 1, 2, 3, 4 and 5) of the benzyl ring A for 4-hydroxypanduratin A and panduratin A (Figure 4) since this ring gave the highest contribution and has many possibilities for substitution. In this work, polar substituents such as –OH, carbonyl group (*i.e*., –COO^−^), nitro group (*i.e*., –NO_2_), amine group (–NH_3_^+^), alkyl group (*i.e*., –CH_3_) and halogen group (*i.e*., –Cl) were placed on positions 1 to 5 of the benzyl ring A. These substituents were added one by one on each position of the ring.

The complexation energies for these newly designed derivatives are presented in Table 2. The derivatives for the ligands substituted at positions 4 and 5 were observed to have complexation energy relatively close to that of 4-hydroxypanduratin A and panduratin A (*i.e.*, −69.8 kcal/mol for 4-hydrxypanduratin A and −57.8 kcal/mol for panduratin A).

Based on this observation, positions (4 and 5) were substituted with other groups and calculated for the complexation energies. Table 3 presented the complexation energies for these newly substituted derivatives.

### 2.3. Synthetic Competitive Dengue Virus Inhibitors

#### 2.3.1. Docking of 246DA, 2446DA and 20H46DA to DEN2 NS2B/NS3

**Figure 5.**
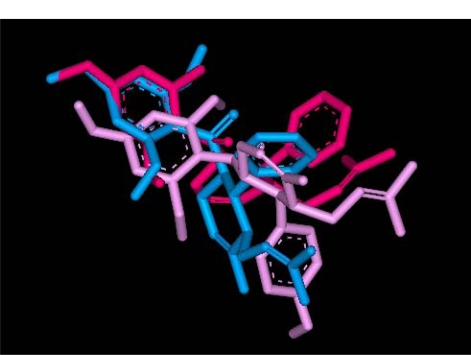
Superimposition of 246DA (blue), 2446DA (pink) and 20H46DA (red).

**Figure 6.**
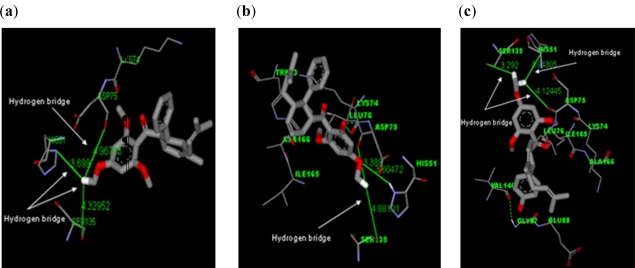
Spatial arrangement of the binding site for (**a**) 246DA (**b**) 20H46DA and (**c**) 2446DA.

Docking of three synthetic compounds onto the catalytic triad of the serine protease enzyme was performed using CHARMM27 force field with a grid box. The best poses from the docking results were selected based on the lowest docked energy values. All three ligands were aligned in a similar manner to natural product compounds (*i.e.*, panduratin A and 4-hydroxypandratin A) in the hydrophilic binding pocket of the protease where the benzyl rings of 246DA, 2446DA and 20H46DA were seen to interact with the active site. The best docked poses of these ligands were superimposed as shown in Figure 5 and the spatial arrangement of the three ligands bound to the active site of the enzyme is presented in Figure 6.

In general, these ligands displayed binding interactions within the active site with the residues as suggested by Bazan and Fletterick [3]. The best docking pose of 246DA (*i.e*., the most active compound) showed more hydrogen bridges with the catalytic triad residues (*i.e*., His51, Asp75 and Ser135) of DEN2 NS2B/NS3 serine protease. 20H46DA and 2446DA were also capable of forming hydrogen bridges with the catalytic triad of this protease but with larger distances. The results (Table 4) indicated the ligand 246DA to be the most active which correlated well with experimental K_i_ value.

The spatial arrangement of the binding site indicated several residues to play important roles in determining the binding interaction for all the ligands. The hydrophobic residues Leu76, Trp83, Ile165 and Ile166 are seen to interact with the ligand through Van der Waals interactions suggesting the importance of these four residues in the formation of Van der Waals specificity pocket. In addition, these four residues also showed electrostatic interaction with the ligands.

#### 2.3.2. Design of New Dengue Virus Inhibitors Based on 246DA, 2446DA and 20H46DA

**Figure 7.**
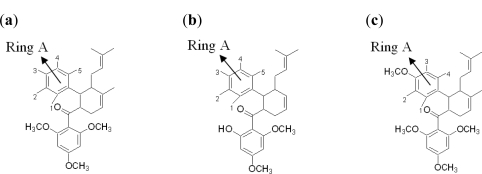
Molecular structures of synthetic inhibitors studied in this work (**a**) 246DA (**b**) 20H46DA and (**c**) 2446DA.

As described earlier, new compounds with potential inhibitory activities against dengue virus were designed by adding substituents to the various positions of the benzyl ring A of 246DA, 224DA and 20H46DA as depicted in Figure 7. In earlier results with 4-hydroxypanduratin A and panduratin A (described above), the greatest contribution in binding with the active site was observed with the substitution on the benzyl ring A. Hence, for this part of the study, we focused only on positions 4 and 5 on the benzyl ring A for 246DA, 20H46DA and 3 and 4 of the benzyl ring A for 2446DA.

Based on the hypothesis that the complexation energy correlates with inhibitory activity, ligands with the lowest complexation energy values and closest to that of the reference compounds are deemed to be the most active. The complexation energies of these new derivatives were computed and the energies obtained were −76.8 kcal/mol, −61.0 kcal/mol and −43.5 kcal/mol for the derivatives of 246DA, 20H46DA and 2446DA, respectively. Table 5 summarizes the complexation energies of the ligand-enzyme complexes when compounds were substituted at positions R and R’.

## 3. Experimental Section

### 3.1. Receptor 3D Structure

The three dimensional structure of DEN2 NS2B/NS3 was downloaded from the protein data bank (http://www.rscb.org/pdb; code 2FOM), the crystal structure was obtained at 1.5 Å resolution. This structure was then minimized using CHARMM27 force field in the MOE software packages (Chemical Computing Group Inc.). The distances of the catalytic triad (*i.e.*, between the carboxyl oxygen of Asp75 and His51 as well as the hydroxyl of Ser135 and His51) were calculated to ensure that they have structural parameters close to the published results [10]. The ligand structures were constructed using ChemDraw (Cambridge software packages) and then imported into MOE software packages (Chemical Computing Group Inc.) to be minimized before beginning the docking process.

### 3.2. Docking of Ligands (Stage 1)

The docking of these five competitive inhibitors, 4-hydroxypanduratin A, panduratin A (Figure 4) and 246DA, 2446DA, 20H46DA (Figure 7) onto the catalytic triad of serine protease were achieved using MOE software packages (Chemical Computing Group Inc.). The docking operation was initiated with the preparation of the protein and the ligands. Hydrogen atoms were added to the serine protease molecule and its backbone was minimized. All ligands were minimized before docking. Docking was performed through simulated annealing algorithm method using CHARMM27 force field (integrated in the MOE software packages, Chemical Computing Group Inc.) with a grid box measuring 26.85 Å × 28.17 Å × 24.53 Å dimension along the *x*, *y*, *z* axes. Upon completion of the docking processes, conformations with the lowest docked energy were chosen and geometry of these enzyme-ligand complexes were minimized to a gradient 0.01 kcal/mol/Å using the same force field. The minimization processes were performed by relaxing the structure step by step as described below. Subsequent minimization with heavy atoms fixed, followed by minimization with the backbone atoms fixed and minimization with the alpha carbons fixed. Finally, minimization of all atoms was carried out.

### 3.3. Computation of Interaction Energy and Binding Energy (Stage 1)

The interaction energies (*i.e.*, Van der Waals and electrostatic energies) and binding energies solvent was not included in computation for binding energy calculation) were then calculated for these complexes. These were carried out using Discovery Studio 2.1 software package (Accelrys). The sum of interaction energy and binding energy is defined as the complexation energy. This energy serves as descriptor in selecting the most active inhibitors.

### 3.4. Selection of Possible Active Inhibitors (Stage 1)

The design of new ligands was based on the shared structure of the said ligands. These new ligands were designed by successive, individual addition of substituents such as the hydroxyl group (–OH), nitro group (–NO_2_), amino group (–NH_3_^+^), carbonyl group (–COO^−^), halogen group (–Cl), and alkyl group (–CH_3_) onto the various positions of the benzyl ring A (positions 1, 2, 3, 4 and 5) as presented in Figures 1 and 2. This stage was only carried out for 4-hydroxypanduratin A and panduratin A. These new complexes were then minimized based on the following steps: addition of substituents to benzene ring followed by fixing of the complex prior to minimization of all the atoms. Subsequently, minimization with heavy atoms fixed, minimization with back bone atom fixed, minimization with alpha carbons fixed and minimization of all atoms were carried out.

The interaction energies, binding energies and complexation energies of these new complexes were then computed. Stage 2 of the design was based on the ligand-enzyme complexes that exhibited the lowest complexation energies close to the values generated by the reference compounds.

### 3.5. Design of New Dengue Virus Inhibitors (Stage 2)

It was observed that the complexes exhibiting the lowest and closest energies to the reference compounds are those substituted at the 4 and 5 positions of the benzyl ring A of 4-hydroxypanduratin A and panduratin A. Thus, in this stage, only these two positions were substituted with various groups (positions 3 and 4 for 2446DA). The complexation energies of these new complexes were computed and compared.

## 4. Conclusions

The docking of two inhibitors extracted from the plant *Boesenbergia rotunda* and three synthetic inhibitors with competitive activities to the DEN2 NS2B/NS3 serine protease were carried out. In this work, the complexation energy of the docking was used as the descriptors for selecting new candidates for competitive dengue inhibitors. The selection was carried out in two stages. In the first stage substitutions were carried out individually on positions 1, 2, 3, 4 and 5 of the benzyl ring A of 4-hydroxypanduratin A and panduratin A. Based on the complexation energies calculated, substitutions at positions 4 and 5 gave the lowest and closest energies to the reference compounds. Subsequently, focus was placed on: positions 4 and 5 for all the reference ligands and positions 3 and 4 in similar location for 2446DA. New ligands were designed by substituting various substituent groups on these positions. Complexation energies for all the new ligand-enzyme complexes were calculated. This strategy reflects a logical progression for early stage drug discovery that can be used to successfully identify drug candidates.

## Figures and Tables

**Table 1.** The complexation energies of 4-hydroxypanduratin A and panduratin A.

**Molecule**	**K_i_ (μM)**	**Complexation Energy (kcal/mol)**
4-hydroxypanduratin	21 ± 8.00 a	−69.8
Panduratin A	25 ± 6.00 a	−57.8

a reference [8].

**Table 2.** Complexation energies of some potential inhibitor derivatives.

**4-hydroxypanduratin A**						

**Position in benzyl ring A**	**Complexation energy of derivatives (kcal/mol)**					
	**–OH**	**–COO^−^**	**–NO_2_**	**–NH_3_^+^**	**–Cl**	**–CH_3_**
1	−37.1	58.1	−1.3	−50.1	−43.1	−38.6
2	−50.4	31.1	240.6	−35.5	−50.7	−44.0
3	−27.1	65.2	−49.3	−63.0	−53.8	−43.9
4	−40.7	−22.5	**−58.4**	**−58.9**	−41.4	−38.5
5	−36.1	**−91.4**	−43.4	**−93.3**	**−51.6**	**−54.1**

**Panduratin A**						

**Position in benzyl ring A**	**Complexation energy of derivatives (kcal/mol)**					
	**–OH**	**–COO^−^**	**–NO_2_**	**–NH_3_^+^**	**–Cl**	**–CH_3_**

1	70.9	13.8	59.9	120.9	54.8	64.4
2	68.4	36.8	−24.3	−14.4	−31.6	−37.9
3	−24.7	5.3	−48.2	−10.7	−62.2	−62.6
4	−51.4	27.2	−40.6	−42.7	**−69.1**	−53.7
5	**−60.8**	−44.7	**−68.0**	**−64.5**	**−55.2**	−54.0

**Table 3.** Some 4-hydroxypanduratin A and panduratin A derivatives substituition at the 4 and 5 positions of the benzyl ring A (R and R’ respectively).

**No**	**Molecule**	**R**	**R’**	**Complexation energy (kcal/mol)**
1	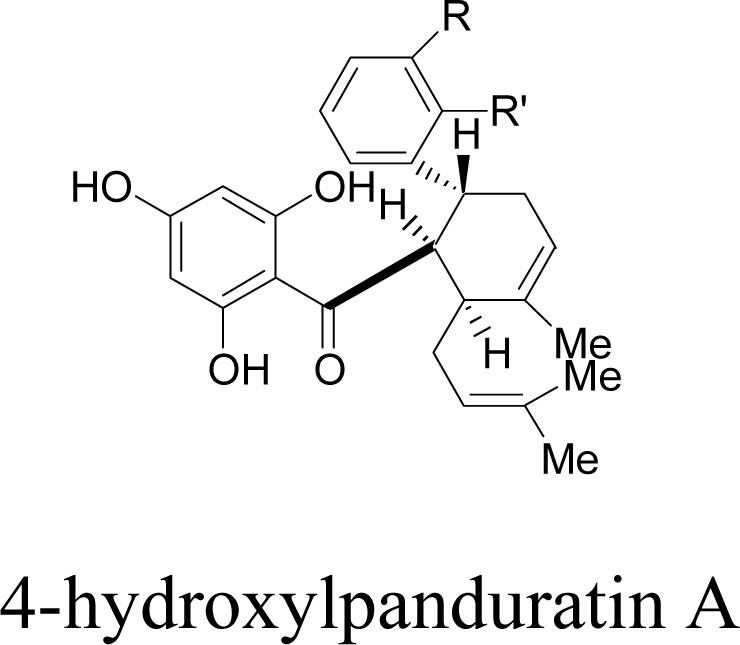	R = NO_2_	R’ = H	−68.7
		R = NH_3_^+^	R’ = H	−68.9
		R = CH_2_NH_3_^+^	R’ = H	−69.7
		R = NH_2_^+^CH_3_	R’ = H	−68.9
		R = H	R’ = COO^−^	−91.4
		R = H	R’ = NH_3_^+^	−93.3
2	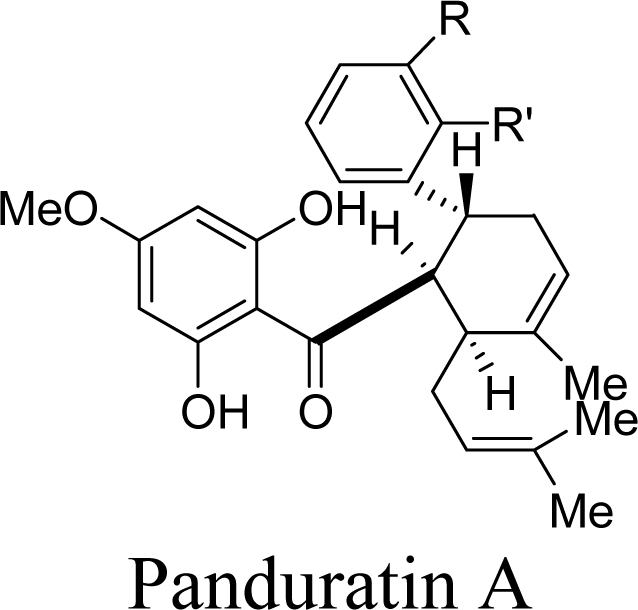	R = Cl	R’ = H	−69.1
		R = OCH_3_	R’ = H	−70.2
		R = SH	R’ = H	−58.8
		R = NH_2_^+^CH_3_	R’ = H	−64.8
		R = H	R’ = OH	−60.8
		R = H	R’ = NO_2_	−58.0
		R = H	R’ = NH_3_^+^	−64.5
		R = H	R’ = Cl	−55.2
		R = H	R’ = CH_2_OH	−66.0
		R = H	R’ = SH	−63.3
		R = H	R’= CH_2_NH_3_^+^	−58.6

**Table 4.** Complexation energies and K_i_ value of the synthetic inhibitors.

**Molecule**	**K_i_ (μM)**	**Complexation Energy (kcal/mol)**
246DA	19.84	−76.8
20H46DA	24.36	−61.0
2446DA	39.68	−43.5

**Table 5.** Some 246DA, 20H46DA derivative substitutions at the 4 and 5 positions and 2446DA derivative substitutions at the 3 and 4 positions of benzyl ring A (R and R’ positions respectively).

**Name**	**Molecule**	**R**	**R’**	**Complexation Energy (kcal/mol)**
246DA	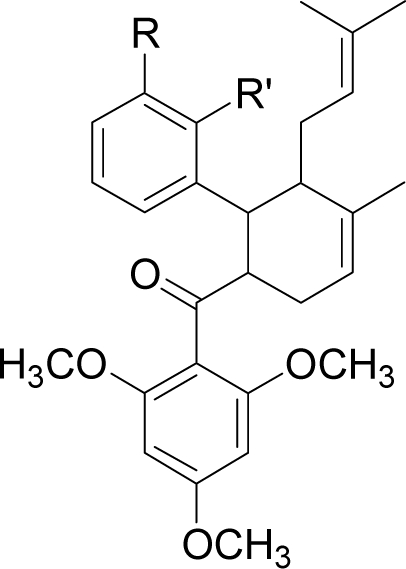	R = OH	R’ = H	−70.2
		R = COO^−^	R’ = H	−117.9
		R = NH_3_^+^	R’ = H	−76.8
		R = OCH_3_	R’ = H	−81.2
		R = CH_2_NH_3_^+^	R’ = H	−74.1
		R = H	R’ = NH_3_^+^	−91.9
		R = H	R’ = CH_2_OH	−90.4
		R = H	R’ = CH_2_NH_3_^+^	−87.6
20H46DA	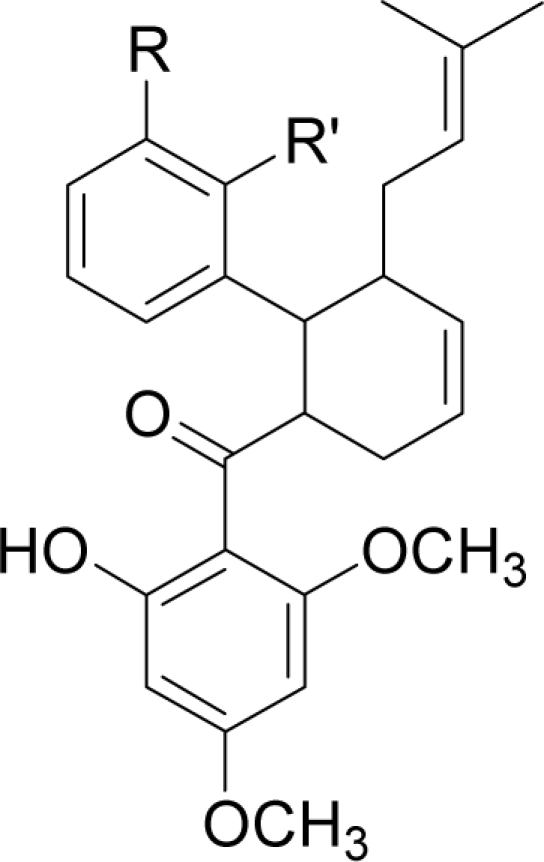	R = OH	R’ = H	−61.6
		R = COO^−^	R’ = H	−61.8
		R = NH_3_^+^	R’ = H	−164.7
		R = CH_3_	R’ = H	−61.0
		R = CH_2_OH	R’ = H	−77.4
		R = H	R’ = COO^−^	−83.5
		R = H	R’ = NO_2_	−64.3
		R = H	R’ = NH_3_^+^	−68.3
		R = H	R’ = OCH_3_	−64.7
		R = H	R’ = CH_2_NH_3_^+^	−129.1
2446DA	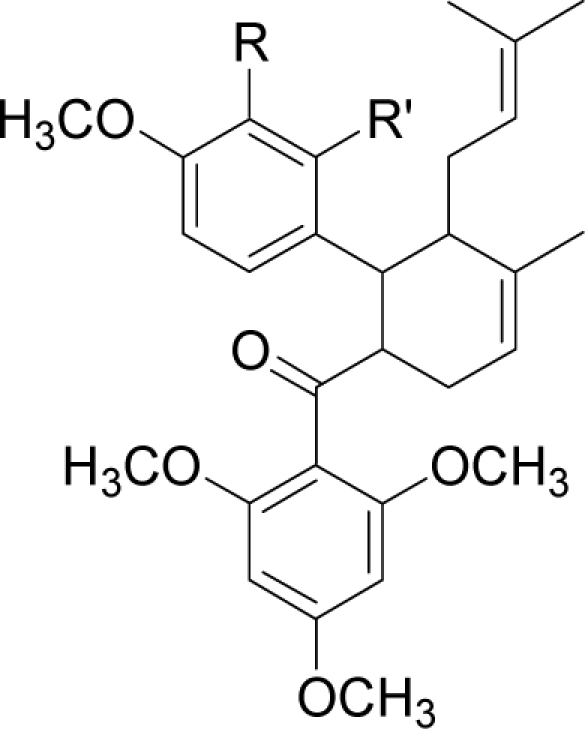	R = H	R’ = OH	−95.3
		R = H	R’ = COO^−^	−108.9
		R = H	R’ = NO_2_	−84.7
		R = H	R’ = NH_3_^+^	−88.9
		R = H	R’ = Cl	−98.7
		R = H	R’ = CH_3_	−87.2
		R = H	R’ = CH_2_OH	−95.7
		R = H	R’ = OCH_3_	−83.8
		R = H	R’ = SH	−73.5
		R = H	R’ = CH_2_NH_3_^+^	−116.7
		R = OH	R’ = H	−88.9
		R = NO_2_	R’ = H	−84.3
		R = NH_3_^+^	R’ = H	−116.5
		R = Cl	R’ = H	−79.1
		R = CH_3_	R’ = H	−76.4
		R = OCH_3_	R’ = H	−81.6
		R = SH	R’ = H	−68.1

## References

[1] Morens D, Fauci A (2008). Dengue and hemorrhagic fever: A potential threat to public health in United States. J. Am. Med. Assoc.

[2] Irie K, Mohan PM, Sasaguri Y, Putnak R, Padmanabhan R (1989). Sequence analysis of cloned dengue virus type 2 genome (new guinea-C strain). Gene.

[3] Bazan JR, Fletterick R (1989). Detection of a trypsin like serine protease domain in flaviviruses and pestiviruses. Virology.

[4] Chambers TJ, Nestorowicz A, Amberg SM, Rice CM (1993). Mutagenesis of the yellow fever virus NS2B protein: Effects on proteolytic processing, NS2B-NS3 complex formation, and viral replication. J. Virol.

[5] Falgout B, Pethel M, Zhang Y, Lai C (1991). Both non structural proteins NS2B and NS3 are required for the proteolytic processing of dengue virus non structural protein. J. Virol.

[6] Leyssen P, de Clercq E, Neyts J (2003). Perspective for the treatment of infections with flaviviridae. Clin. Microbiology.

[7] Sampath A, Padmanabhan R (2009). Molecular targets for flavivirus drug discovery. Antivir. Res.

[8] Tan SK, Pippen R, Yusof R, Ibrahim H, Khalid N, Rahman NA (2006). Inhibitory activity of cylohexenyl chalcone derivatives and flavanoids of fingerroot, *Boesenbergia rotunda* (L.), towards dengue-2 virus NS3 protease. Bioorg. Med. Chem. Lett.

[9] Yean KL, Othman R, Wahab HA, Yusof R, Rahman NA (2006). A revisit into the DEN2 NS2B/NS3 virus protease homology model: strcutural verification and comparison with crystal structure of HCV NS3/4A and DEN2 NS3. Malays J Sci.

[10] Yean Kee L, Tan SK, Wahab HA, Yusof R, Rahman NA (2007). Non substrate based inhibitors of dengue virus serine protease: A molecular docking approach to study binding interactions between protease and inhibitors. Asia Pacific J. Mol. Biol. Biotechnol.

[11] Brinkworth RI, Fairlie DP, Young PR (1999). Homology model of the dengue 2 virus NS3 protease: putative interactions with both substrate and NS2B cofactor. J. Gen. Virol.

